# Doctoral School Day in Public Health: Liège, Belgium. 13 November 2018

**DOI:** 10.1186/s13690-019-0333-5

**Published:** 2019-02-28

## Health promotion

### A1 - Understanding turning points in the process of changing attitude towards the practice of Female Genital Mutilation among migrant women in Belgium

#### Afi Agboli^1^*, Fabienne Richard^2,3^, Olivier Schmitz^1^, Isabelle Aujoulat^1^

##### ^1^Research Institute Health and Society (IRSS), Université Catholique de Louvain, Brussels, Belgium; ^2^GAMS, Brussels, Belgium; ^3^Université Libre de Bruxelles, Brussels, Belgium

###### **Correspondence:** Afi Agboli (afi.agboli@uclouvain.be)

**Background:** The change of any norm is difficult especially if it is a social norm deeply rooted in the traditions and practises of a whole community. It is the case of female genital mutilation (FGM), a social norm and harmful practice where women are not only victims, but they are also the perpetrators. However, some women have changed their attitude towards the practice. This study seeks to explore how the change occurs. Thus, it investigates significant events in the trajectories of the migrant women in relation to their change of attitude.

**Methods:** Fifteen women with FGM living in Belgium were recruited through gatekeepers and snowball procedure. They were met twice for individual interview using biographical narrative interview method. Congruently with this interview method, we did not have a structured interview guide. The analysis drew on a life story approach and lifeline constructions to identify significant and common turning points (TP) by which the change occurs.

**Results:** Six important TP were identified as factors of awareness leading to a change of attitude and in relation to a challenge of related norms: *change in the perception of pain; sense of responsibility to protect daughters; confrontation with the anatomy of an intact female external genitalia; awareness through the confrontation with other women’s cultures; awareness that they are not defined exclusively by FGM. The change in the representation of pain during sexual intercourse*, for example, was considered normal in the home country but was no longer perceived as normal in Belgium by most research participants. This TP challenged the norm *‘women must endure pain and suffering’.*

**Conclusions:** Professionals working with women who have undergone FGM may use the TP to give awareness in order for other women to take action and change their attitude towards the practice.

**Keywords:** FGM, migrant women, turning points

### A2 - How to tackle nursing students’ self-esteem decline? A qualitative phenomenological study

#### Jacinthe Dancot*, Benoit Pétré, Michèle Guillaume

##### Department of Public Health, University of Liège, Liège, Belgium

###### **Correspondence:** Jacinthe Dancot (jacinthe.dancot@uliege.be)

**Background:** Self-esteem is proved to have a significant impact on nurses’ professional behaviour and competence. Previous studies have highlighted that nursing students reported a lower self-esteem than the general population of other students. Moreover, other reports have pointed out a decrease in self-esteem during the education process of nurses. The aim of this study was to explore the key factors and mechanisms involved in the self-esteem of nursing students.

**Methods:** A purposive sample of 41 (33 females and 8 males) first- and second-year nursing students from 4 Belgian Colleges was interviewed in order to investigate perceived self-esteem and potential influencing factors selected from an integrative literature review. A phenomenological thematic analysis was done using NVivo 12 and themes were assembled in an explicative model following Mruk’s theory (2013).

**Results:** Students described their self-esteem during the first two years of education as being highly unstable. Mruk explains such variations through what he calls ‘self-esteem moments’, which are related to various stress factors including (1) frequently having to be accepted in new groups (as during training periods) and (2) rapidly and recurrently having to prove their competence. Students described two moments mostly impacting their self-esteem: (1) the training period and particularly the nursing teams’ and teachers’ attitudes; and (2) receiving their exam results. Students reported that such ‘self-esteem moments’ influenced their professional behaviour, leading to either proactive engagement, or defensive withdrawal. The level of engagement fostered more or less the competence development, forming a circle either virtuous or vicious.

**Conclusion:** These first results allow to identify the perceived critical ‘self-esteem moments’ at the beginning of nursing curriculum and to explore their consequences on students’ self-esteem and engagement. Based on these results, actions focusing on self-esteem moments and on students’ ability to cope with them could be suggested to nursing schools.

**Keywords:** self-esteem, nursing student, clinical competence

### A3 - Knowledge, attitude and practices of pregnant women and gynaecologists-obstetricians regarding omega-3 polyunsaturated fatty acid consumption during pregnancy

#### Axelle Hoge^1^*, Florence Bernardy^1^, Anne-Françoise Donneau^1^, Nadia Dardenne^1^, Sylvie Degée^2^, Michelle Nisolle^2^, Michèle Guillaume^1^, Vincenzo Castronovo^3^

##### ^1^Department of Public Health, University of Liège, Liège, Belgium; ^2^Department of Obstetrics and Gynaecology, CHR Citadelle Hospital, University of Liège, Liège, Belgium; ^3^Metastasis Research Laboratory, GIGA-CANCER, University of Liège, Liège, Belgium

###### **Correspondence:** Axelle Hoge (axelle.hoge@uliege.be)

**Background:** Omega-3 polyunsaturated fatty acids (n-3 PUFA) during pregnancy have been subject to large media coverage, early and non-definitive research on health benefits and absence of consensual guidelines for the management of nutrient deficit. These conditions have shown to create a situation of confusion among both patients and healthcare providers. This cross-sectional study was carried out to explore knowledge, attitude and practices regarding n-3 PUFA in two independent populations of pregnant women and gynaecologists-obstetricians. The relationship between pregnant women’s attributes and their n-3 PUFA status measured by the omega-3 index (IOM3) was also investigated.

**Methods:** Participants included 122 women in early pregnancy and 67 gynaecologists-obstetricians. Knowledge, attitude and practices were collected by self-administered questionnaires. Fasting blood specimens were obtained from each pregnant woman at recruitment for testing for IOM3. The IOM3 was defined as erythrocyte eicosapentaenoic plus docosahexaenoic acids expressed as weight percentage of total fatty acids.

**Results:** Marked discrepancies in perception were observed between the pregnant women and the gynaecologist-obstetricians. While 82% of the women gave high importance to n-3 PUFA during pregnancy, only a third did so among health providers. About 35% of the women declared paying particular attention to their n-3 PUFA intake. After adjusting for sociodemographic characteristics, these favourable dietary practices were significantly associated with higher omega-3 index (p=0.04). Overall 43.3% of the professionals didn’t provide any information about n-3 PUFA to their pregnant patients and 46.3% didn’t implement any preventive actions when suboptimal n-3 PUFA levels were suspected.

**Conclusions:** Evidence-based guidelines, refreshment training and communication tools are needed to improve awareness and clinical practices among health providers regarding n-3 PUFA, aiming ultimately at health benefit for both mothers and their children.

**Keywords:** omega-3 fatty acids, perception, practices, pregnancy, healthcare professionals

### A4 - Male partners’ information needs, information seeking behaviour, and decision-making during the first time pregnancy of their female partners in Nigeria: a qualitative study

#### Chiemeka Onyeze-Joe^1^*, Celine Mahieu^2^, Isabelle Godin^2^

##### ^1^Université Libre de Bruxelles, Brussels, Belgium; ^2^Research Centre in Social Approaches to Health, School of Public Health, Université Libre de Bruxelles, Brussels, Belgium

###### **Correspondence:** Chiemeka Onyeze-Joe (conyeze@ulb.ac.be)

**Background:** Male partners experiencing the pregnancy of their female partners for the first time in Nigeria are often ignorant about what they need to do to encourage a safe and healthy maternity. As key decision makers in the traditional family, male partners can ensure maternal access to resources and support if informed. This study explores the information seeking behaviour, preference of sources and priorities of first-time fathers that influence decision making.

**Methods:** In this qualitative study, 50 men whose partners have been pregnant within the last two years were recruited from hospitals, public places, churches and recreational centres in rural and urban settings in south-east Nigeria. An interview guide was developed from ideas provided from similar studies in sub-Saharan Africa, with three main questions in mind: what are the information needs of the male partners? how do male partners seek information regarding pregnancy? and how is decision making influenced by their information needs? Data were collected through semi-structured interviews, and the analysis was performed following Braun & Clarke’s 6-step method: 1) read data to become familiar with them, 2) develop initial codes, 3) look for patterns and group them into categories and sub categories 4) review categories in relation to coded extracts 5) generate clear names for each theme 6) write-up.

**Results:** The male partners had similar information needs including financial cost of care and safety of the mother and child, but these needs were prioritised based on their education level, perception of gender roles, location and beliefs. Their decision making regarding the place of delivery was influenced the most by the availability of finances and trust in the professional experience of caregivers.

**Conclusion:** This study identifies several information needs and avenues to focus on when designing intervention strategies that reach out to male partners to improve their willingness to be involved in pregnancy related care in Nigeria.

**Keywords:** Information needs, first-time fathers, pregnancy

## Epidemiology

### A5 - Comparing administrative and survey data for ascertaining chronic disease prevalence

#### Finaba Berete^1,2^*, Stefaan Demarest^1^, Jean Tafforeau^1^, Herman Van Oyen^1,3^, Olivier Bruyère^2^, Johan Van der Heyden^1^

##### ^1^Sciensano, SD Epidemiology and public health, Brussels, Belgium; ^2^Department of Public Health, Epidemiology and Health Economics, University of Liège, Liège, Belgium; ^3^Department of Public Health, Ghent University, Ghent, Belgium

###### **Correspondence:** Finaba Berete (Finaba.Berete@sciensano.be)

**Background** Chronic diseases (CDs) prevalence is often estimated using surveys, disease registers or outpatient records. In the absence of these, administrative databases such as health insurance data can be used. However, the accuracy of case ascertainment in administrative databases is a concern. The aim of this study is to compare prevalence estimates of CDs in Belgium, using the Belgian Mandatory Health Insurance data (BEMAHI) with those obtained in the Belgian Health Interview Survey (BHIS).

**Methods:** Individual BHIS 2008 data were linked with BEMAHI data (n=10,828) using the national register number. CDs in the BEMAHI data were ascertained using previously validated case definitions based on billing information of “disease-specific” drugs (ATC codes). Self-reported CDs were ascertained using a single question “Have you had (name of CD) in the past 12 months?”. Weighted prevalence rates from both data sources were available for 7 CDs. Agreement was measured by estimating sensitivity, specificity, positive and negative predictive values (PPV, NPV) and Cohen's kappa statistic (κ) using BHIS data as the reference.

**Results:** BEMAHI prevalence rates were close BHIS estimates. Agreement was good for diabetes, Parkinson’s disease and thyroid disorders (κ range: 0.62-0.77), moderate for cardiovascular diseases (κ=0.58), fair for epilepsy, asthma and chronic obstructive pulmonary diseases (COPD) (κ range: 0.27-0.38). Sensitivity was moderate to high except for COPD (32.8%) and asthma (32.1%). The specificity was higher than 90% for all the CDs.

**Conclusions** For several CD, prevalence estimates based on billing data yield similar results as those obtained in a health survey. For CDs which are usually treated with specific medications prevalence estimates based on billing data can be an acceptable alternative. However, if the CD is not unambiguously treated with medication, the risk of misclassification is high, which is for instance the case for asthma and COPD.

**Keywords**: chronic diseases, linkage, prevalence, administrative data

### A6 - Adverse events associated with symptomatic slow-acting drugs in osteoarthritis (SYSADOAs): A systematic review and stratified meta-analysis of randomised, double-blind, placebo-controlled trials

#### Germain Honvo^1,2^* (germain.honvo@uliege.be)

##### ^1^Department of Public Health, Epidemiology and Health Economics, University of Liège, Liège, Belgium; ^2^WHO Collaborating Centre for Public Heath Aspects of Musculoskeletal Health and Aging, Liège, Belgium

**Background**: The SYSADOAs are an important drug class in the treatment armamentarium for osteoarthritis (OA). While some are considered safe, some concerns have been raised about the safety profile of others. We aimed to further assess the safety of various SYSADOAs in the management of OA.

**Methods:** We performed a systematic review and random-effects meta-analysis of placebo-controlled trials evaluating oral SYSADOAs in patients with OA. The databases Medline, CENTRAL and Scopus were searched. Adverse events (AEs) related to various System Organ Classes were investigated, along with total, overall severe and serious AEs and withdrawals due to AEs. Authors or sponsors of studies were contacted for full safety reports.

**Results:** An initial 25 studies that did not allow concomitant anti-OA treatments during the trial were selected, in accordance with the protocol. An additional 37 studies with concomitant anti-OA treatments (mainly NSAIDs) were included in parallel analyses. Ultimately, 13 and 18 studies respectively had adequate data for meta-analyses. Glucosamine sulfate (GS), Chondroitin sulfate (CS) and Avocado/soybean unsaponifiables (ASU; Piascledine®) were not associated with increased odds of AEs, compared to placebo. Patients who received Diacerein had 2.53 times (OR 95% CI: 1.43-4.46) more gastrointestinal and 3.16 times (OR 95% CI: 1.93-5.15) more urinary disorders (urine discolouration), than those on placebo. In studies with concomitant anti-OA treatments, diacerein was associated with more withdrawals due to AEs (OR: 3.18; 95%CI: 1.85-5.47) and more skin disorders (OR: 2.47; 95% CI: 1.42-4.31). No significant increase in severe or serious AEs was found.

**Conclusions:** GS and CS can be considered safe for patients with OA. All eligible studies on ASU allowed other anti-OA medications; however, current evidence seems to support its safety. Diacerein should be considered taking into account the nature of the product, its dosage, and patient characteristics.

**Keywords**: Adverse events – Glucosamine sulfate – Chondroitin sulfate – Diacerein - Avocado/soybean unsaponifiables

### A7 - The effect of the use of PRISMA on the reporting quality of meta-analyses

#### Victoria Leclercq^1^*, Charlotte Beaudart^1^, Sara Ajamieh^1^, Véronique Rabenda^1^, Ezio Tirelli², Olivier Bruyère^1^

##### ^1^Department of Public Health, Epidemiology and Health Economics, University of Liège, Liège, Belgium; ^2^Department of Psychology, University of Liège, Liège, Belgium

###### **Correspondence:** Victoria Leclercq (victoria.leclercq@uliege.be)

**Background:** Recent analyses of published Meta-analyses (MAs) suggest that their methodological quality is suboptimal. However, robust MAs are essential for researchers, clinicians and policy makers to make decisions. Subsequently, the methodological quality of MAs can only be assessed if all needed information is well reported in the manuscript. Our aim is to investigate the effect of the explicit use of PRISMA *(Preferred Reporting Items for Systematic reviews and Meta-analyses)* on the reporting quality of MAs.

**Methods:** We evaluated a random sample of 207 MAs indexed in PsycINFO database in 2016 of which 100 explicitly used PRISMA and 107 MAs did not. Two authors have independently extracted data on reporting quality using the 27 items of PRISMA.

**Results:** The overall median adherence to PRISMA was significantly different in the two groups (PRISMA=85%, No PRISMA=70%, p<0.0001). In fact, 12 out of the 27 items of the PRISMA statement were significantly more encountered in the PRISMA group than in the no PRISMA group (i.e. structured summary, protocol and registration, information sources, search strategy, study characteristics, results of individual studies, funding and, in both methods and results sections, study selection, risk of bias in individual study and bias across studies (all p-values are <0.05)). In the PRISMA group, for example, the probability to have information about protocol and registration (OR: 10.95, IC95%:3.18-37.67), the risk of bias in individual studies in either the methods (OR: 3.88; IC95%:2.18-6.92) or the results (OR: 5.75; IC95%:3.15-10.49) sections or the search strategy (OR: 4.18; IC95%:2.34-7.51) were significantly higher than in the no PRISMA group.

**Conclusions:** The explicit use of PRISMA has a positive influence on the reporting quality of MAs. Even in MAs using PRISMA, reporting is not optimal. The use of PRISMA should be more widespread to facilitate the critical assessment of validity of the published Mas.

**Keywords:** Meta-analysis, Reporting, Quality, PRISMA statement, Meta-research

### A8 - Three-year adverse health consequences of sarcopenia in community-dwelling older adults according to five diagnosis definitions

#### Médéa Locquet^1^*, Charlotte Beaudart^1^, Jean Petermans^2^, Jean-Yves Reginster^1^, Olivier Bruyère^1^

##### ^1^Department of Public Health, University of Liège, Liège, Belgium; ^2^Geriatrics Department, CHU of Liège, Liège, Belgium

###### **Correspondence:** Médéa Locquet (medea.locquet@uliege.be)

**Background**: Sarcopenia is considered a public health burden, because of its association with adverse consequences. Our aim was to assess the association between sarcopenia and adverse health consequences over a 3-year period in community-dwelling elders using 5 different definitions of sarcopenia.

**Methods**: The SarcoPhAge (for *Sarcopenia and* *Physical Impairment with advancing* *Age*) project includes 534 older subjects. Sarcopenia was clinically defined as low muscle mass plus low grip strength and/or decreased physical performance, and variations were observed between cut-off limits of each component for 5 operational definitions. Data on adverse outcomes were collected yearly during a visit or a phone call. The association between sarcopenia and the occurrence of outcomes was tested using Cox hazards model, when survival data were available. If not, this was tested using logistic regression model. The scenario was repeated for each definition.

**Results**: Five hundred thirty-four subjects were recruited into this prospective study (73.5±6.2 years, 60.5% female). After 3 years of follow-up, data were then available for 501 subjects. If no association between baseline sarcopenia and physical disabilities or institutionalisations was highlighted, a higher number of deaths occurred in individuals with sarcopenia than in those healthy (16.2% versus 4.6%, p-value<0.001) for the European Working Group on Sarcopenia in Older People (EWGSOP) definition. The 3-year probability of death when presenting sarcopenia showed an approximately 3-fold increase compared to subjects without (4 out 5 definitions). A longer duration of hospital stay was observed in subjects with sarcopenia when defined using the EWGSOP or by the Asian Group, but no association was observed between sarcopenia and falls or fractures.

**Conclusion**: Sarcopenia was associated with an increased risk of mortality and with longer hospital stays. There were variations in the ability of different definitions to predict these outcomes.

**Keywords:** Sarcopenia, Mortality, Consequences, Prospective study, SarcoPhAge

### A9 - Malaria risk assessment at local level using satellite imagery and Boosted Regression Trees (BRT) in the Peruvian Amazon

#### Elisa Solano-Villarreal^1,2,3^*, Walter Valdivia^4^, Catherine Linard^5,6^, J. J. Pasapera-Gonzales^7^, Philippe Lejeune^1^, Alejandro Llanos-Cuentas^2^, Niko Speybroeck^2^, Marie-Pierre Hayette^1^, Angel Rosas-Aguirre^2,3^

##### ^1^University of Liège, Liège, Belgium; ^2^Universidad Peruana Cayetano Heredia, Lima, Perú; ^3^Université Catholique de Louvain, Brussels, Belgium; ^4^Ministerio de economía y Finanzas, Lima, Perú; ^5^Université de Namur, Namur, Belgium; ^6^Institute of Life-Earth-Environment (ILEE), Namur, Belgium; ^7^Comisión Nacional de Desarrollo Aeroespacial, Lima, Perú

###### **Correspondence:** Elisa Solano-Villarreal (elitayoan@gmail.com)

**Background:** Malaria in Loreto department remains a public health problem, accounting for more than 90% of reported cases in Peru. This is the first study in the Peruvian Amazon aimed at assessing the risk of malaria transmission using satellite imagery and Boosted Regression Trees (BRT).

**Methods:** Villages with at least one malaria case between 2010 and 2015 from the routine surveillance data in Loreto were georeferenced and their cases aggregated by year and species. Social and environmental variables were derived from Landsat satellite imagery and other spatial data, then included as explanatory variables into a cross-validated Poisson BRT model for malaria incidence at the local level. Time-dependent explanatory variables included forest coverage (FC, %), annual forest loss (FL,%), cumulative annual rainfall (CAR, mm), annual-mean land surface temperature (LST, ^o^C), normalised difference vegetation index (NDVI), and normalised difference water index (NDWI). Other variables were Euclidean shortest distance to rivers (SDR, meters), time to major populated villages/towns (TPV, minutes), and night-time lights (NTL, mean value 2010-2013) as proxy of population density. BRT accounts for nonlinearities and interactions between factors with high predictive accuracy for disease risk mapping.

**Results:** A total of 1524 villages were included in the analysis (70% of total Loreto’s villages). More than 90% of relative influence in the overall malaria incidence was explained by five variables: NTL (67.8%), TPV (8.1%), FC (6.5%), CAR (5%) and SDR (4.6%). The analysis by species showed a higher influence of environmental variables (CAR, LST, NDVI and NDWI) for *P. falciparum* (18.4%) than for *P. vivax* incidence (9.7%). Malaria risk maps were generated based on model predictions taking into account the relative influence of variables.

**Conclusions:** Remotely sensed data analysed using BRT allowed for maps delimiting areas of high malaria risk in Loreto. These maps will help malaria stakeholders to prioritise areas for control interventions.

**Keywords:** Malaria risk, Satellite Imagery, Boosted Regression Trees, Peruvian Amazon

## Health system

### A10 - Costs of inpatient stays with liver diseases directly related to alcohol: primary results

#### Caroline Delo*, Philippe Van Wilder, Pol Leclercq, Magali Pirson

##### Centre de recherche en Economie de la Santé, Gestion des Institutions de Soins et Sciences Infirmières, Ecole de Santé Publique, Université Libre de Bruxelles, Brussels, Belgium

###### **Correspondence:** Caroline Delo (carodelo@ulb.ac.be)

**Background:** In 2018, the World Health Organisation (WHO) published alarming statistics showing that excessive alcohol consumption is still prevalent, and estimated that alcohol causes more than 3 million deaths each year (28% were due to injuries, 21% due to digestive disorders, 19% due to cardiovascular diseases). This study evaluates the costs (for the hospital, for the social security and for the patient) of inpatient stays with liver diseases directly related to alcohol.

**Methods:** The selection criterion is the principal diagnosis grouped in K70- Alcoholic liver disease [ICD 10CM classification]. We studied 9 independent variables from a medical and administrative database, and 4 cost variables from the “Projet d’Analyse de Coût des Hôpitaux Associés” (PACHA) database. The median comparisons were tested using the Mann Whitney-Wilcoxon test.

**Results:** The sample population studied consists of 749 inpatient stays, in 15 French-speaking hospitals in Belgium. The sample represents 0.3 % of total inpatient stays (excluded day hospitalisations). The median cost (minimum-maximum) for the hospital for these inpatient stays is € 4,306.54 (€27.55 - €89,633.69), and the median length of stay is 7.39 days (0.40 days – 131.57 days). 59 % of the median cost is related to the hospitalisation costs. Seven independent variables (on 9) significantly influence the costs [severity index of illness, type of admission, destination after the hospitalisation, etc.]. Indeed, the patients with a hospitalisation in the intensive care unit have a median cost of € 17,900.45. By contrast, the patients who are not hospitalised in this unit have a median cost of € 3,687.89 (p<0.005).

**Conclusion:** The first results of this study suggest that many variables significantly influence the cost of inpatient stays. Various results in this article may be of interest to hospital managers in a context of hospital financing reform.

**Keywords:** Alcohol, cost, hospital, social security, liver disease

### A11 - Development of an instrument to measure paediatric palliative care outcomes: a pilot-study

#### Marie Friedel^1,2^*, Bénédicte Brichard^3^, Sabine Boonen^4^, Corinne Tonon^4^, Brigitte de Terwangne^4^, Jean-Marie Degryse^1,5^, Isabelle Aujoulat^1^

##### ^1^Institute of Health and Society (IRSS), Université Catholique de Louvain, Brussels, Belgium; ^2^Institut Parnasse-Institut Supérieur d'Enseignement Infirmier, Haute Ecole Léonard de Vinci, Brussels, Belgium; ^3^Department of Pediatric Hemato-oncology, Cliniques Universitaires St Luc, Université Catholique de Louvain, Brussels, Belgium; ^4^Interface pédiatrique, Cliniques universitaires Saint-Luc, Université Catholique de Louvain, Brussels, Belgium; ^5^Department of Public Health and Primary Care, Katholieke Universiteit Leuven, Leuven, Belgium

###### **Correspondence:** Marie Friedel (marie.friedel@uclouvain.be)

**Background:** Paediatric palliative care (PPC) aims to promote quality of life (QoL). However, French validated short instruments able to measure multidimensional components of children’s QoL in a palliative context and a family approach are still lacking. A promising, but not yet validated, instrument in English, called Children’s Palliative Outcome Scale (CPOS Downing 2012, 2018), was found through literature review. The CPOS is a standardised questionnaire evaluating PPC outcomes for children and their parents, combining self and proxy report. The objective of the pilot-study was to further develop the CPOS, translated in French, by testing it among children receiving palliative care, their parents and health care professionals (HCP).

**Methods:** After translating the CPOS into a French version, semi-structured interviews among children and their parents, in presence of a HCP were organised. Two instruments, the CPOS and the quality of life in life threatening illness-family caregiver questionnaire (QOLLTI-F, Cohen 2006, 2015) and one interview guide (the Scheduled Evaluation of Individual Quality of life, SEIQOL Hickey 1998) were used during the interviews. The content-validity of the CPOS was assessed by comparing the dimensions explored by the original CPOS with the emerging dimensions raised by the use of the SEIQoL and the QOLLTI-F.

**Results:** Overall, 14 interviews were conducted. Some emerging dimensions regarding social, emotional, financial and administrative issues were added to the original English version of the CPOS, leading to a modified multidimensional including 12 questions addressed to the child (self-report or proxy report) and 10 questions to the parents.

**Conclusion:** Through this pilot-test, an adapted French version of the CPOS was made possible in a collaborative process between children receiving palliative care, their parents and the HCP.

**Keywords:** Children, outcomes measures, palliative care, pilot-test, quality of life

### A12 - Impact of the 2015 earthquake in hospital admissions at the Tribhuvan University Teaching Hospital: preliminary results

#### Maria Moitinho de Almeida^1^*, Benjamin Schluter^1^, Sunil Thapa^2^, Joris van Loenhout^1^, Ravikant Singh^3,^ KC Kumar^2^, Deepak Mahara^2^, Debarati Guha-Sapir^1^

##### ^1^Centre for Research on the Epidemiology of Disaster (CRED), Institute of Health and Society, Université Catholique de Louvain, Brussels, Belgium; ^2^Tribhuvan University Teaching Hospital, Kathmandu, Nepal; ^3^Doctors for you, Mumbai, India

###### **Correspondence:** Maria Moitinho de Almeida (maria.rodrigues@uclouvain.be)

**Background:** In April 2015, Nepal was hit by a 7.8 magnitude earthquake, causing nearly 9,000 dead and 22,000 injured. The Tribhuvan University Teaching Hospital (TUTH) in Kathmandu received a high share of people affected. Literature on earthquake impact on hospitals is scarce, particularly in the mid and longer term.

**Methodology:** To understand the effect of the earthquake in the functioning of TUTH, we gathered admission data from 1.5 months before to 3 months after the earthquake. We collected sociodemographic and clinical information (ICD-10 codes), and admission and discharge dates of patients. We defined 4 periods: Pre-Earthquake (PREEQ), Acute Earthquake Period (3 weeks after the first shake – EQ1), Post-Acute Earthquake Period (3 weeks after the acute period – EQ2), and Post Earthquake (POSTEQ). We performed descriptive analysis and logistic regression with period as outcome variable, taking sex and diagnosis in consideration.

**Results:** A total of 9615 admissions were included. In EQ1, and compared to PREEQ, the odds of admission due to injury had the highest significant increase compared to other diagnoses (OR= 5.36, p<0.001). There was a significant decrease in the odds of admission of 0-4 age group compared to the 15-49 group (OR=0.68, p<0.01). In EQ2, the odds of being admitted due to respiratory diseases, and pregnancy-related issues were significantly lower relative to PREEQ (OR=0.770, p<0.05; OR=0.78, p<0.05 respectively). Finally, in POSTEQ, the odds were lower for injuries (OR=0.84, p<0.05), pregnancy (OR=0.72, p<0.001), and people aged 50 or more (OR=0.85, p<0.01), relative to PREEQ.

**Discussion:** The higher odds of admission for injuries in the acute period is supported by the literature. The decrease in pregnancy-related admission implies that women’s access to this hospital was reduced. This study supports a conceptual model proposed of hospital needs after a sudden disaster, where trauma emergencies increase in a very short-term, leaving non-trauma emergencies and elective admissions for a post-acute phase.

**Keywords:** earthquake, patient admissions, disaster

### A13 - “Patient participation” and related concepts: a scoping literature review on their dimensional composition

#### Iness Ortiz Halabi^1^*^†^, Isabelle Bragard^1†^, Nancy Durieux^2^, Béatrice Scholtes^1^, Bernard Voz^1^, Nicolas Gillain^1^, Olivier Ziegler^3,4^, Remy Gagnayre^5^, Michèle Baumann^6^, Angela Odero^6^, Benoit Pétré^1†^, Michèle Guillaume^1†^

##### ^1^Department of Public Health, University of Liège, Liège, Belgium; ^2^Library of Life Sciences, Department of Speech Therapy, University of Liège, Liège, Belgium; ^3^LORDIAMN Regional Network, Nancy, France; ^4^Laboratory of Nutrition, University of Lorraine, Nancy, France; ^5^Laboratory of Health Education and Practice (LEPS), University of Paris 13, Paris, France; ^6^Research Institute of Sociology and Economy of Inequalities (IRSEI), Belval Campus, Esch-sur-Alzette, Luxembourg

###### **Correspondence:** Iness Ortiz Halabi (iness.ortiz@ulg.ac.be)

^#^jointly co-authors

**Background:** Healthcare systems are going through a change, in which patients have been expected to be more involved participants in their care. Several concepts on the collaboration between patients and healthcare systems have emerged in the literature but there is little consensus on their meanings and differences. In this study, the generic concept of “patient participation” was studied by considering all of the related concepts equally and by focusing on the dimensions that compose them. This review follows two objectives: (1) to produce a detailed and comprehensive overview of the “patient participation” dimensions; (2) to identify differences and similarities between the related concepts.

**Methods:** A scoping review of the literature was performed to review and synthesise knowledge into a conceptual framework. A summative process was used to collect the data that was classified in three levels of analysis: micro level (day-to-day management of care), meso level (hospital governance and institutional decisions) and macro level (government decisions, organisation and funding of the healthcare sector). Finally, a thematic analysis was used to analyse the data.

**Results:** The thematic analysis resulted into a detailed description of the concept of “patient participation” at the three levels of analysis. Twenty-nine dimensions composing the generic concept of “patient participation” partnership (such as teaching patients, sharing leadership and decision making, partnership care, patient engagement continuum, healthcare professionals and patient training, and patient participation in research); 5 influencing factors (such as patients’ and healthcare professionals’ socio-demographic and psychosocial background); and 4 expected outcomes (such as better health ourcomes and improved healthcare system) were identified.

**Conclusion**: This global vision of “patient participation” allows to go beyond the oppositions between the existing concepts. A consensus on this matter needs to be agreed upon in order for “patient participation” to be implemented.

**Keywords:** patient participation, patient empowerment, patient centred-care, literature review, thematic analysis.

### A14 - Impact of psychiatric hospitalisations on the social integration of patients with severe mental illness: a study in five European countries

#### Pierre Smith, Pablo Nicaise, Vincent Lorant & the COFI study group

##### Institute of Health and Society (IRSS), Université Catholique de Louvain, Brussels, Belgium

###### **Correspondence:** Pierre Smith (pierre.smith@uclouvain.be)

**Background:** During the last three decades, a decrease in psychiatric hospital admissions and a reduction in the length of stay (LoS) occurred in high-income countries, with a view to promote community care and the social integration of patients with severe mental illness (SMI). However, the impact of hospitalisations on patients’ social integration remains unclear. The aim was to determine (1) whether readmissions and longer LoS in psychiatric wards decreased the social integration of SMI patients and (2) which are the most affected dimensions of social integration.

**Methods:** Within the European COFI study, data were collected for 2009 SMI patients hospitalised in 2015 in the UK, Italy, Germany, Poland and Belgium. Social integration was measured using the SIX index at baseline and at one year of follow-up. The SIX index includes four dimensions: employment, housing, living situation and friendship. Pearson and spearman correlations and multiple regression models were performed to test the association between LoS, the number of admissions, and the change in patient’s social integration over a year.

**Results:** There was a significant negative correlation between LoS (r_s_ = -0.06, p < 0.01), the number of admissions (r_s_ = -0.04, p = 0.04) and the change of patients’ social integration over a year. After controlling for patients’ clinical and sociodemographic variables, an increase of the total LoS was significantly associated with a decrease of the employment (OR = 1.19, p = 0.02) and housing (OR = 1.95, p < 0.01) status levels, but not the number of readmissions.

**Conclusions:** Lengthy LoS in psychiatric ward have a more negative impact on SMI patients’ social integration than repeated admissions. Housing and employment are the main dimensions of social integration negatively associated with LoS. Therefore, special attention should be paid to helping patients find and retain housing and employment during psychiatric hospitalisations.

**Keywords**: Mental illness, Social integration, Length of hospital stay, Hospital readmissions

### A15 - Through patients and professionals representations on patient partnership of care: the exploration of an ambiguous consensus – Preliminary results

#### Bernard Voz*, Iness Ortiz Halabi, Benoît Pétré, on behalf of the consortium APPS

##### Department of Public Health, University of Liège, Liège, Belgium

###### **Correspondence:** Bernard Voz (bernard.voz@uliege.be)

**Background:** Patient engagement in health care system has been regarded as a lever for the general improvement of the health system. Many models illustrate and participate in this change. One of them proposes a patient-partnership, challenging the whole health system, from direct care to policy making. In a context where the Belgian health system is put into question, the relevance of patient participation has to be challenged. This exploratory study is aimed at exploring the representations, experiences and expectations towards these models. Through this contribution, we will highlight the understanding of a partnership in health for patients and professionals.

**Methods:** As part of a larger Interreg research project, a qualitative approach has permitted an in-depth comprehension of patients and hospital professionals representation toward the partnership model. A purposive sample of thirty patients, suffering from cancer, diabetes, cardiovascular or pulmonary diseases, has been met in six focus groups. Thirty professionals, nurses and specialist physician in charge of chronic patients, have taken part in semi-structured interviews. Thematic analysis was used to organise the data.

**Results:** Patients and professionals share common basis for partnership, perceiving it as positive and, in some way, already happening. Both patients and professionals mention, among other things, the importance of common language, listening skills and team work. Beyond these discourses, some divergences appear when considering what partnership could mean in practice. As an example, information sharing is a key debate for the actors, who don’t agree about what should be shared, how or with whom. Furthermore, if patients and professionals easily identify what a partnership should be in direct care, they struggle to conceive one at other levels.

**Conclusions:** This analysis urges the health actors to face their divergence of opinions about interdisciplinarity, knowledge, and the sharing of information. These results constitute an initial trigger for the development of grounded interventions.

**Keywords:** Patient partnership, health system, patient participation

## Simulation in healthcare

### A16 - Validation of an immersive virtual reality environment for mass casualty incidents to enhance health care professionals training

#### Manon Goosse^1^*, Jean-Christophe Servotte^1,2^, Bruno Pilote^3^, Ivan L. Simoneau^4^, Suzanne Campbell^5^, Michèle Guillaume^1^, Alexandre Ghuysen^1,2^, Isabelle Bragard^1,2^

##### ^1^Department of Public Health, University of Liège, Liège, Belgium; ^2^Department of Public Health, Interdisciplinary Medical Simulation Centre of Liège, University of Liège, Liège, Belgium; ^3^Nursing Department, Sainte-Foy College, Québec, Québec, G1V 1T3, Canada; ^4^Nursing Department, Cégep de Sherbrooke, Sherbrooke QC, J1E 4K1, Canada; ^5^School of Nursing, the University of British Columbia, Vancouver BC, Canada

###### **Correspondence:** Manon Goosse (manon.goosse@uliege.be)

**Background:** Whereas Mass Casualty Incidents (MCI) occur with a growing incidence, health care professionals are not prepared to address those disasters that involve high levels of stress. MCI training is imperative but limited mainly due to logistical and cost problems involved by actual training methods. Virtual reality could address those issues. This study aims to validate the quality of an MCI virtual environment (MCI-VE) simulating a school car accident in a tunnel.

**Methods:** The MCI-VE was created by a multi-disciplinary team following the standards of good practice in simulation (Jeffries, 2015; Lioce et al. 2015). It aims to train technical (e.g. choosing the right place for the ambulance, triage, lifesaving intervention) and non-technical skills (e.g. stress management, communication) required in MCI management. To validate this environment, it was tested with a group of undergraduates (UG, 42 undergraduate medicine and 3^rd^-year nursing students) and of specialised professionals (SPG, 19 fourth-year nursing students specialising in ICU). Several factors were assessed with questionnaires at two times, before (immersion propensity, stress) and after immersion (sense of presence, stress, cyber sickness and satisfaction) in both groups.

**Results:** Level of presence was high among both groups, but higher among SPG (p = 0.02), and was positively correlated to immersion propensity (r=0.36; p<0.01). Both groups reported low levels of cyber sickness. There was a significant interaction effect between group and time for stress (p *<* 0,001): SPG’s stress increased between the two assessment times whereas it decreased in the UG. 100% of students reported that the experience helps the learning.

**Conclusion:** Our study brings first data to validate this MCI-VE. Further studies have to test its impact on skills learning, before to consider it as an alternative to current pedagogical methods.

**Keywords:** Virtual reality training – Mass casualty incidents – Technical and non-technical skills

### A17 - Immersion Clinical Simulation (ICS): what is a real Impact on knowledge acquisition?

#### Bruno Pilote^1^*, Christophe Chenier^2^, Jean-Christophe Servotte^3^, Ivan L. Simoneau^4^

##### ^1^Nursing Department, Sainte-Foy College, Québec, Québec, G1V 1T3, Canada; ^2^Pedagogy and Education Department, UQAM, Montréal, Canada; ^3^Department of Public Health, University of Liège, Liège, Belgium; ^4^Nursing Department, Cégep de Sherbrooke, Sherbrooke QC, J1E 4K1, Canada

###### **Correspondence:** Bruno Pilote (bruno.pilote.1@ulaval.ca)

**Background:** For several decades, ICS has been one of various teaching strategies aimed at increasing students’ knowledge contain. To this day, the impact of ICS on knowledge acquisition cannot be fully ascertained because there are a lot methodological limitations: students’ self-reporting, identical pretest and post-test examinations, use of a single post-test, or absence of a control group. However, all of these situations affect the methodological quality of the studies and theirs conclusions. Indeed, no studies have compared the impact of ICS on knowledge acquisition from two group when perform the equivalent but not similar Multiple Choice Question (MCQ) exam.

**Methods:** This prospective, multicentre study is based on a quasi-experimental research. The participants in the experimental group were taught using a series of four progressive ICS including debriefing session in addition to internship, while those in the control group were taught using internship alone. Before testing, we developed and validated two similar exams about cardiology knowledge with a RASH method. The knowledge of the participants was assessed twice based on MCQ about their cardiology knowledge: version A in pretest conditions (before internship and ICS) and version B in post-test conditions. Each MCQ, about cardiology knowledge, consists of 35 items, including seven common items.

**Results:** A total of 177 nursing students (N=177) were involved in this research project, including 93 (n=93) in the experimental group and 84 (n=84) in the control group. Under pretest conditions, the results obtained by the two groups on version A of the exam questionnaire were found statistically equivalent (p=.63). Under post-test conditions, participants in the experimental group scored significantly higher (p=.002).

**Conclusion:** The results of this research further more confirm the impact of simulation on knowledge acquisition.

**Keywords**: Clinical immersion simulation, knowledge acquisition

### A18 - Prehospital assessment of trauma patients: impact of a high-fidelity simulation training among last year nursing students

#### Jean-Christophe Servotte*, Isabelle Bragard, Jérémy Renard, Alexandre Ghuysen

##### Department of Public Health, Interdisciplinary Medical Simulation Centre of Liège, University of Liège, Liège, Belgium

###### **Correspondence:** Jean-Christophe Servotte (jcservotte@uliege.be)

**Background:** In Europe, nearly 152 000 deaths occur each year following a trauma. By improving prehospital care, a reduction of this number is possible. At the scene of the accident, nurses assess patients with the Airway, Breathing, Circulation, Disability, and Exposure primary assessment (ABCDE) and secondary assessment. While these assessments seem of paramount importance to ensure patient safety, nurses are not always well prepared for it and can present a lack of knowledge and skills. Simulation could be an effective pedagogical method for improving these skills.

**Methods:** This randomised controlled trial is aimed at assessing the impact of a high-fidelity simulation-based learning in trauma management on self-efficacy, knowledge and initial assessment skills. Last year, nursing students were randomised to a training-group (TG, n = 25) or to a waiting-list-group (WLG, n = 22). TG received a pre-briefing with two prehospital trauma assessment videos, followed by a 2-hour high-fidelity simulation. WLG participated in the usual course implying a 1-hour theoretical course and a 1-hour skills lab. Before (T1) and after (T2) training, nursing students were assessed with questionnaires and a prehospital trauma high-fidelity simulation. Their skills during simulation were rated with the National Registry of Emergency Medical Technicians (NREMT) trauma patient assessment checklist. Questionnaires were used to assess knowledge, self-efficacy and self-reported stress.

**Results:** Repeated measures ANOVA revealed a difference of evolution from groups over the time on prehospital assessment skills (P < 0.001), self-efficacy (P = 0.001) and knowledge (P = 0.03). TG improved skills by 248% whereas WLG improved these by only 68%.

**Conclusions:** A short simulation-based learning has the potential to significantly improve pre-hospital assessment skills. These results advocate the interest of such training in nursing curriculum.

**Keywords:** simulation-based learning; high-fidelity; trauma-patients; prehospital assessment

### A19 - Development of a competency framework for polytrauma patients’ management in the Greater Region

#### Jean-Christophe Servotte^1^*, Pierre-Marie Waselle^1^, Pauline Van Ngoc^1^, Tobias Fritz^2^, Tim Pohlemann^2^, Tina Histing^2^, Alexandre Ghuysen^1^, Isabelle Bragard^1^

##### ^1^Department of Public Health, Interdisciplinary Medical Simulation Centre of Liège, University of Liège, Liège, Belgium; ^2^Saarlandes University, Saarbrücken, Germany

###### **Correspondence:** Jean-Christophe Servotte (jcservotte@uliege.be)

**Background:** The Greater Region (GR) is Europe’s cross-border region including Lorraine, Luxembourg, Rhineland-Palatinate, Saarland and Wallonia. These regions share a consortium founded by five Universities. In the GR, emergency initial training for polytrauma patients’ management is heterogeneous. Moreover, learners encounter difficulties in acquiring knowledge and skills on trauma due to case limitations. This lack of experience can lead to adverse events and care errors. Lastly, WHO requests that states of the European Union introduce training in prehospital trauma care. Consequently, the five universities are designing a common competency framework to define technical and non-technical skills needed to manage polytrauma patients.

**Methods:** To design this competency framework, several steps have been planned: 1) a literature review on Medline Databases and CINAHL using key words related to training and polytrauma management, and consultation of reference books; 2) a first proposal of common frame; 3) face-to-face and experts’ meetings; and 4) a Delphi method to finalise and validate the framework. The first two have already been carried out.

**Results:** The literature review allowed selecting two books and 23 articles. The first proposal of competency framework is divided into three parts. The first part presents prerequisite knowledge and skills of the emergency physicians. The second part describes the rational of the frame, and essential concepts as the Golden hour^6^ and a timeline for polytrauma patients’ management. The last part describes seven medical skills using CanMEDS system presented in two sections: five Non-Technical Skills (Professional attitude, Long-life learning, Communicator, Collaborator and Leader), and several Technical Skills to use during Prehospital phase and In-hospital phase.

**Conclusions:** Designing a common framework for polytrauma patients’ management in the GR is the first step of an Interreg project. Based on CanMEDS system, seven skills have been defined. A Delphi method will validate this proposal.

**Keywords:** framework; polytrauma; management

### A20 - European Project SimuCarePro-CRM: simulation in healthcare and crisis resource management to increase the efficiency of multidisciplinary teams in initial training

#### Jean-Christophe Servotte^1^*, Laurence Peeters^2^, Méryl Hajaoui^1^, Zlata Selak^3^, Amina Ouersighni^5^, José Amado Martins^4^, Riu Batista^4^, Claudia Gherman^6^, Alexandre Ghuysen^1^, Isabelle Bragard^1^

##### ^1^Department of Public Health, Interdisciplinary Medical Simulation Centre of Liège, University of Liège, Liège, Belgium; ^2^Haute Ecole Libre Mosane, Liège, Belgium; ^3^Inforef, Liège, Belgium; ^4^University of Coïmbra, Coïmbra, Portugal; ^5^Paris Descartes University, Paris, France; ^6^Cluj-Napoca University, Cluj-Napoca, Romania

###### **Correspondence:** Jean-Christophe Servotte (jcservotte@uliege.be)

**Background:** In Europe, 8 to 12% of patients suffer adverse events, 70%of which have a non-technical origin (diagnosis error, inefficient communication, etc.). Consequences of those communication errors can be a worsening clinical state, appearance or worsening of irreversible sequelae, a longer stay at the hospital, etc. International organisations recommend developing in healthcare professionals’ initial training non-technical skills, gathered under the term “Crisis Resource Management” (CRM).

**Methods:** SimuCarePro-CRM is an Erasmus+ project led by Helmo Paramedical Department, and funded by the European Union gathering training organisations from Belgium, France, Portugal and Romania. It is aimed at developing a European training program for non-technical skills addressed to medicine and nursing care students. Two educational methods will be used: e-learning and clinical simulation. The project will last for two years and will include six steps: 1) current situation of the concept of CRM and CRM training; 2) development of an e-learning platform; 3) creation of e-learning-based training modules; 4) production and validation of CRM-focused scenarios; 5) implementation of the training activity and 6) evaluation and formulation of recommendations.

**Results:** A digital and interactive theoretical training in CRM will be freely accessible to teachers, students and healthcare professionals. The simulation scenarios specific to each CRM component will be available in open access. The added value of multidisciplinary CRM learning will be tested using evaluation grids for non-technical skills and satisfaction questionnaires submitted to students from four countries.

**Conclusions:** This innovative European project aims to develop and implement a CRM training. It should help improve future healthcare professionals’ skills and, eventually, patients’ safety. The efficiency of the project will be monitored by self-efficacy and satisfaction questionnaires as well as competency assessment.

**Keywords:** CRM, training, skills

